# Nurses’ perceptions of a pressure ulcer prevention care bundle: a qualitative descriptive study

**DOI:** 10.1186/s12912-016-0188-9

**Published:** 2016-11-21

**Authors:** Shelley Roberts, Elizabeth McInnes, Marianne Wallis, Tracey Bucknall, Merrilyn Banks, Wendy Chaboyer

**Affiliations:** 1 NHMRC Centre of Research Excellence in Nursing, Menzies Health Institute Queensland, Griffith University, Gold Coast Campus, Queensland, 4222 Australia; 2 Nursing Research Institute, St Vincent’s Health Australia (Sydney) and School of Nursing, Midwifery and Paramedicine, Australian Catholic University, North Sydney, NSW 2060 Australia; 3 School of Nursing, Midwifery and Paramedicine, University of the Sunshine Coast, Locked Bag 4, Maroochydore DC, QLD 4558 Australia; 4 Centre for Quality and Patient Safety, School of Nursing and Midwifery, Deakin University, Geelong, VIC 3220 Australia; 5 Department of Nutrition and Dietetics, Royal Brisbane and Women’s Hospital, Herston, QLD 4019 Australia

**Keywords:** Care bundle, Evidence-based practice, Implementation science, Knowledge translation, Nurses, Patient engagement, Patient participation, Pressure injury prevention, Pressure ulcer prevention, Process evaluation

## Abstract

**Background:**

Pressure ulcer prevention is a critical patient safety indicator for acute care hospitals. An innovative pressure ulcer prevention care bundle targeting patient participation in their care was recently tested in a cluster randomised trial in eight Australian hospitals. Understanding nurses’ perspectives of such an intervention is imperative when interpreting results and translating evidence into practice. As part of a process evaluation for the main trial, this study assessed nurses’ perceptions of the usefulness and impact of a pressure ulcer prevention care bundle intervention on clinical practice.

**Methods:**

This qualitative descriptive study involved semi-structured interviews with nursing staff at four Australian hospitals that were intervention sites for a cluster randomised trial testing a pressure ulcer prevention care bundle. Four to five participants were purposively sampled at each site. A trained interviewer used a semi-structured interview guide to question participants about their perceptions of the care bundle. Interviews were digitally recorded, transcribed and analysed using thematic analysis.

**Results:**

Eighteen nurses from four hospitals participated in the study. Nurses’ perceptions of the intervention are described in five themes: 1) Awareness of the pressure ulcer prevention care bundle and its similarity to current practice; 2) Improving awareness, communication and participation with the pressure ulcer prevention care bundle; 3) Appreciating the positive aspects of patient participation in care; 4) Perceived barriers to engaging patients in the pressure ulcer prevention care bundle; and 5) Partnering with nursing staff to facilitate pressure ulcer prevention care bundle implementation.

**Conclusions:**

Overall, nurses found the care bundle feasible and acceptable. They identified a number of benefits from the bundle, including improved communication, awareness and participation in pressure ulcer prevention care among patients and staff. However, nurses thought the care bundle was not appropriate or effective for all patients, such as those who were cognitively impaired. Perceived enablers to implementation of the bundle included facilitation through effective communication and dissemination of evidence about the care bundle; strong leadership and ability to influence staff behaviour; and simplicity of the care bundle.

## Background

Pressure ulcer prevention (PUP) is a significant priority in hospitals given the high incidence of pressure ulcers (PUs) [1], the severe consequences they have for patients [2], and the large costs they incur to the health care system [3, 4]. Nurses have a primary role in PUP; however patients may also contribute through active participation in PUP care [5, 6]. Patient participation in care has been shown to result in improved patient safety [7] and satisfaction with care [8]. Patient participation is endorsed by the World Health Organisation [9] and is included in health service standards internationally [10, 11]. Nurses partnering with patients in PUP may be an effective strategy for reducing pressure ulcers (PU) among at-risk individuals.

Our team developed a pressure ulcer prevention care bundle (PUPCB) targeted at both patients and nurses, encouraging patient participation in PUP care with three simple evidence-based messages: 1) Keep moving; 2) Look after your skin; and 3) Eat a healthy diet [12]. These messages were delivered to patients with a poster, brochure and DVD. Nurses received training on how to partner with patients in PUP by encouraging active participation in their care. The effectiveness of the care bundle for PUP was tested in a cluster randomised trial, the INTroducing A Care bundle To prevent pressure ulcers (INTACT) trial, in eight Australian hospitals [13]. Hospitals were randomly assigned to the intervention (PUPCB) or control group. Medical, surgical and rehabilitation wards considered to have a high number of patients at risk of PU were included in the study.

The INTACT trial showed a significant reduction in PU incidence in the intervention group at the hospital (cluster) level, but this difference was not significant at the individual patient level. Whilst there are many possible reasons for these findings, the research team concluded the study was underpowered [13]. Given this, it seems logical to further investigate the processes relating to the intervention itself and its use. The success or failure of interventions, or the extent to which they work, may in part be explained by processes relating to implementation [14]. Hence, a pre-specified process evaluation was conducted alongside the INTACT trial to give insight into the trial’s findings. Process evaluations provide an understanding to assist with future implementation of interventions, recognising that moving research into routine practice is difficult [15]. Grant et al.’s framework for process evaluations of cluster randomised trials [16] guided our evaluation.

This paper reports in detail on the response of nursing staff (clusters) to the intervention; one domain of the process evaluation framework. It is important to understand nurses’ experience with and perceptions of an intervention; particularly one that involves nurses partnering with patients in care. Their perspectives of the intervention and the evidence around it, the context in which it used, and characteristics of nurses themselves are likely to influence its adoption in practice and ultimately, its effect [17]. This study aimed to explore nurses’ perceptions of a PUPCB, including its impact and how it might be incorporated into usual practice, to give insights into the main trial findings, wider PUPCB implementation, and translation into routine practice.

## Methods

### Study design and setting

This qualitative descriptive study [18] involved semi-structured interviews with nursing staff working on wards where the PUPCB was implemented, in four acute care hospitals (public and private) that were intervention sites for the cluster randomised trial across two Australian states. Ethical approval was gained at each hospital intervention site and from the participating university.

### Participants and recruitment

Nurses of any designation working on the INTACT study wards who were (a) permanent full time or part time employees; and (b) employed during the INTACT trial were eligible to participate in the study. With assistance from the nurse unit manager, potential participants meeting inclusion criteria were identified. Purposive sampling was used to recruit four to five nurses per site, depending on the point at which data saturation was reached, to include a mix of nurses in terms of gender, area of speciality and years’ experience in nursing. All nurse participants were provided with a participant information sheet and written informed consent was gained from those agreeing to participate. The number of participants was determined when data saturation was reached (i.e. data collection ceased when no new information was identified).

### Data collection

A semi-structured interview guide was developed based on published methodology [16, 19] and piloting of the intervention [20]. An iterative process of reviewing, piloting and refining the interview guide was undertaken, involving senior members of the research team with experience in acute care, hospital-based nursing and qualitative research. Questions were structured within four main domains: (1) Awareness of the INTACT trial and intervention; (2) Perceptions of the intervention; (3) Perceived impact of the intervention; and (4) Utility of the PUPCB in usual practice. Questions and prompts for each domain are shown in Table 1.

**Table 1:** Semi-structured interview domains and questions

Domain	Example questions and prompts
1) Awareness of the INTACT trial and intervention	• What was the purpose of the INTACT trial? • What were the main messages you took from the INTACT trial?
2) Perceptions of the intervention	• What was good/bad about the PUPCB? •What did you think of the poster/brochure/DVD?
3) Perceived impact of the intervention	• Were there any benefits or limitations for your unit being involved in the INTACT study? Why/why not? • Were staff affected in any way? How?
4) Utility of the PUPCB in usual practice	• Do you think this intervention/care bundle should be adopted by the ward for all patients at risk of PU? Why/why not? • What barriers or enablers do you see for using this in practice?

Note: Generic prompts included: ““Can you tell me more about this?”; “Can you explain this further / expand on this?” and “What do you mean when you say ____?”

Individual interviews were conducted on site by a trained research assistant, at a time and location that was convenient for the participant. A conversational style of interviewing was used with the semi-structured interview guide providing direction for the interviewer. Direct questions were asked when conversations did not elicit responses about the PUPCB. Interviews lasted approximately 15–20 min and were digitally recorded and later transcribed for analysis.

### Data analysis

Thematic analysis was used to analyse the semi-structured interview data [21, 22]. Interview transcripts were read and re-read; codes were developed based on participants’ verbatim statements; and data relevant to each code were collated into sub-themes and themes based on commonality. Analysis was led by one member of the research team, with close conferral with another team member and input from the whole research team to clarify findings. Trustworthiness of findings was enhanced by: (a) discussing findings among the research team to ensure data were interpreted in line with the emergent codes, sub-themes and themes (credibility); and (b) using purposive sampling to ensure broad representation of nurses (transferability) [23].

## Results

Eighteen nurses participated in interviews across the four hospitals (at least four nurses from each site). The majority of nurses were female (*n* = 15, 83%). Nurses came from a mix of medical, surgical and rehabilitation wards. Participants’ experience in nursing ranged from 3 to 30 years. No participants who were approached declined to participate.

Nurses answered questions about their perceptions of the intervention related to the domains outlined above. Their responses were then able to be grouped into five themes: 1) Awareness of the PUPCB and its similarity to current practice; 2) Improving awareness, communication and participation with the PUPCB; 3) Appreciating the positive aspects of patient participation in care; 4) Perceived barriers to engaging patients in the PUPCB; and 5) Partnering with nursing staff to facilitate PUPCB implementation. Nurses spent longer responding to questions about their perceived impact of the intervention and the utility of the PUPCB in practice (i.e. domains 3 and 4), which was reflected in the themes. The themes and their sub-themes are outlined in Table 2 and described in further detail below.

**Table 2:** Themes and sub-themes

Themes	Sub-themes
Awareness of the PUPCB and its similarity to current practice	Understanding the PUPCB
	Comparing the PUPCB and current practice
Improving awareness, communication and participation with the PUPCB	Increasing awareness of PUP
	Enhancing nurse and patient communication about PUP
	Active participation in PUP
Appreciating the positive aspects of patient participation in care	Understanding and supporting patient participation in care
	Realising the benefits of patient participation
Perceived barriers to engaging patients in the PUPCB	Cognitive impairment restricts participation in PUP
	Taking a passive approach to health care
	Undervaluing of PUP by patients
Partnering with nursing staff to facilitate PUPCB implementation	Communication and dissemination
	Leadership and influence
	Keeping the PUPCB simple

### Awareness of the PUPCB and its similarity to current practice

All nursing staff were aware of the INTACT trial happening on their ward. Most nurses had heard about the study either at an in-service or from a colleague. In this theme, nurses described their perceptions of the intervention (PUPCB), including their understanding of what it involved and how they thought it compared with their current practice.

#### Understanding the PUPCB

Most nurses had a good understanding of the PUPCB’s purpose (PUP); key concepts (nurse-patient partnership and patient participation); and messages (keep moving, look after your skin, eat a healthy diet). Most nurses said they had not seen the intervention materials in much detail but generally, those that had gave positive feedback about the components of the PUPCB, stating that they were simple and easy for patients to understand.

*…patient involvement in their own care for preventing pressure injuries. And also, staff engaging with the patients so it’s a more collaborative approach to looking after the patients. (Site 2, N7)*

*Educating the patients to help themselves as well, so to move more, and healthy diet… and to work with staff was a big part of it as well. (Site 2, N8)*

#### Comparing the PUPCB and current practice

Nurses discussed the PUPCB in terms of their current practice. Some suggested their practice already reflected the PUPCB, so it didn’t add anything new to nursing practice on that ward. For this reason some nurses expressed doubt that the PUPCB would work. Others were more accepting of the PUPCB, recognising it could assist in PUP.

*I’m not really sure if it will work for orthopaedic because the patients are bed ridden and we check their skin regularly ourselves. So I’m not really sure if it would be really beneficial. (Site 3, N14)*

Others however, favourably compared the PUPCB to their current practice, describing how it aligned with other initiatives or strengthened what they were already doing in practice.

*It did tie in very nicely with what we’re trying to do already, which is a lot more patient involvement. (Site 2, N7)*

### Improving awareness, communication and participation with the PUPCB

Nurses described the potential benefits of the PUPCB for both patients and staff, discussing several changes they believed occurred on the ward; including increased awareness of PUP, improved nurse and patient communication about PUP and active participation in PUP.

#### Increasing awareness of PUP

Nurses expressed there was increased awareness of PUP amongst both patients and staff as a result of the intervention. They believed the intervention raised nurses’ awareness of PU, PUP and patients who may be at risk of PU. One nurse believed the PUPCB messages aimed at patients also resonated with staff, stating that it encouraged nurses to consider patients’ nutritional status, skin care and mobilisation in everyday care.

*It was beneficial, because it raised awareness not only amongst the patients that were chosen to be part of the trial…. but awareness of staff as well. (Site 3, N13)*

*I guess it makes you think more about who’s at risk. (Site 4, N15)*

Participants thought nurses became more aware of the concept of patient participation, which facilitated nurses to allow patients to be more independent in their care.

*It made us nurses aware as well….because when you’re in a hurry you tend to do things for them [patients]. But it made you…stop and think, and make them [patients] more independent to take control of what they’ve got to do. (Site 3, N12)*

Participants perceived that after receiving the PUPCB, patients became more interested in and aware of PU, and had a better understanding of what they needed to do in terms of PUP activities and why they needed to do it.

*They [patients] become curious about pressure sores and who gets them and how do you get them and how common they are. (Site 2, N6)*

#### Enhancing nurse and patient communication about PUP

Nurses thought the intervention resulted in improved communication amongst patients and staff (i.e. patient-to-nurse, nurse-to-nurse, and patient-to-patient interactions) around PUP. Nurses said patients were more often engaging with them in conversations about repositioning, including asking if they needed to be turned or communicating to staff that they were moving themselves. Nurses thought the intervention made it easier to start conversations with patients about PUP.

*It actually helped the staff to generate the conversations (about PUP) with the patient. (Site 3, N4)*

Nurses also expressed the PUPCB helped patients understand why nurses implement certain interventions such as repositioning. They stated that often patients did not like moving themselves or being repositioned by nursing staff due to pain or immobility, but once patients understood what was happening and why, they were more compliant and less anxious with being repositioned or moving themselves.

*Rather than us nagging them constantly, they’re kind of aware why we want to roll them. (Site 2, N6)*

The intervention was believed to have generated conversations about PUP amongst staff and encouraged a team approach to PUP. One nurse described how she witnessed a patient conversing with another patient about what he had learned from the PUPCB.

*Another patient who had just had the DVD shown to him ended up having a conversation with the patient across from him…..he started showing the patient the brochure from his bedside, saying ‘here, this is what they told me’ and ‘this is the poster they put up on the wall’ and he started telling him about what he was just taught. I was quite surprised as to how much he retained and what he actually had a conversation with the other patient. (Site 3, N13)*

#### Active participation in PUP

Nurses explained how they thought the intervention encouraged more active participation in PUP by patients; particularly in terms of moving. They believed patients became more independent, active and interested in moving; needing less direction and encouragement to move. Nurses described how patients often rolled or repositioned themselves without being asked; or, if they needed help repositioning, they were more likely to ask.

*Most of the patients that I was taking care of that received that information… were actively changing their positions without being told to do so. (Site 1, N4)*

*Some people got really into it. And they’d say things like ‘do I need to be turned?’ and things like that. (Site 1, N3)*

### Appreciating the positive aspects of patient participation in care

Nurses had overwhelmingly positive views of patient participation in care. Appreciation took two forms including understanding and supporting participation; and realising the benefits of patient participation in care.

#### Understanding and supporting patient participation in care

Nurses explained how they understood patient participation in care, including what they thought it meant in theory and what activities it involved in practice. They described aspects of participation such as shared responsibility and knowledge/information exchange, and also acknowledged considerations such as patients’ abilities and level of engagement when involving them in their care. They discussed their personal views on and experiences with patient participation, with most nurses advocating an active role for patients in care.

*The more information we can provide to the patient to start taking some form of responsibility right from admission throughout their journey, I think that’s imperative. We need to give some of the responsibility back to them as well. (Site 3, N13)*

*It’s good for them to have an awareness that they’re responsible and they are playing a part in their own health care and their processes of getting… discharged out of hospital. (Site 2, N7)*

#### Realising the benefits of patient participation

Nurses described many perceived benefits of patient participation in their health care to both patients and staff. Nurses expressed when patients participate in their care, this provides ‘whole care’ and patients experience better outcomes. They thought patients had an improved mindset about their own care and were more satisfied with their hospital stay when they participated in care. They also thought that participation seemed to improve a patient’s mood.

*It’s good if they participate as well because it’s a whole care. Rather than for us to keep implementing but there will be no effect, because it’s up to the patient to agree with the treatment or with the implementation. So it’s very good if they know exactly what we are doing for them and they know the benefits and they can help us in that way. (Site 1, N2)*

Another perceived benefit of patient participation was that it sometimes saved work for nurses. For example, participants conveyed that when patients are taught to participate in their own care, they are enabled to care for themselves proactively without being asked or reminded.

*If we teach the patients on how to take control of their own care, then we can enable them to do it more proactively than waiting on the nurses to tell them what to do and everything. (Site 1, N4)*

Nurses also thought sharing more information with patients was beneficial for both patients and health care professionals, as patients usually reciprocated by providing important information back to the nurse or being more willing to participate in care.

*…the more information you share, the more education that you give them, they actually help you. And in return you get what you need, which is the optimum outcome which is for them to be discharged with good health or get into the right frame of mind about their own health care. (Site 2, N7)*

### Perceived barriers to engaging patients in the PUPCB

Nurses suggested that whilst the PUPCB may work for some, it was not appropriate for all patients as it required active participation and engagement, to which there were many barriers. Three sub-themes were evident: ‘Cognitive impairment restricts participation in PUP’, ‘Taking a passive approach to health care’ and ‘Undervaluing of PUP by patients’.

#### Cognitive impairment restricts participation in PUP

Many nurses believed that cognitively impaired patients, who may be at higher risk of PU, were unable to benefit from this intervention. Patients with dementia, delirium or confusion were described as being very high risk, but being unable to participate in PUP activities due to limited ability to self-care and being unable to understand or retain information provided in PUP education. It was interesting that nurses did not acknowledge differences in patients’ abilities to participate depending on their stage of dementia (i.e. early or advanced dementia). However, nurses did often mention a solution to this barrier, which was involving the family or carers in the patient’s care. Educating carers and family members around PU risk factors and PUP strategies was seen to be extremely important for patients with cognitive impairment.

*If they’re a person with failing memory or a bit of dementia, I don’t know about its [PUPCB] effectiveness or their ability to retain the information. However, if they’re a patient who has family visiting it’s good to educate the family as well I believe. At least the family are more aware so when they come and visit… they may be on the lookout for these things more often. (Site 3, N11)*

#### Taking a passive approach to health care

Nurses discussed how some patients had a passive approach or negative attitude towards their health care, which was perceived as a barrier to participation. They said some patients didn’t believe it was their responsibility to participate, thought nurses should do everything for them, would not take ownership of their care, and assumed the ‘sick role’ whilst in hospital. Nurses did not think all patients were willing to participate in their care and acknowledged this as a limitation. One nurse mentioned that in these cases, a solution may also be to involve the family in the patient’s care.

*….it works for a certain percentage….. it doesn’t work for everyone. It depends on the person… whether or not people believe it’s their responsibility to take part in that role and I think for some patients it’s a bit difficult for them to take ownership of what their responsibilities are. (Site 2, N7)*

#### Undervaluing of PUP by patients

Another barrier to patient participation in PUP care mentioned by nurses was patients’ perceived lack of importance of PUP. This may be due to failing to understand one’s own PU risk, or having other issues that patients prioritise higher than PUP. Nurses said that some patients, especially those who were younger or had never had a PU, may not acknowledge they are at risk of developing a PU. They also stated that patients who were bedridden, in pain, or too unwell may not realise the importance of PUP, or were unlikely to prioritise this in terms of other health issues they were experiencing in hospital

*You’d say to them you need to start moving but they wouldn’t really because they’re like, ‘I’m young’; but they still do get red bottoms and red heels. (Site 2, N9)*

*But unless they’ve had a pressure sore they’re not really aware of how easily it can happen. (Site 2, N6)*

### Partnering with nursing staff to facilitate PUPCB implementation

Most nurses thought the PUPCB tested in the INTACT trial should be adopted by their ward as usual practice. Participants highlighted how important it was that the research team partnered with nursing staff to successfully implement the care bundle into practice, and discussed a number of perceived barriers and enablers to implementation.

#### Communication and dissemination

Nurses expressed the importance of communicating information and disseminating evidence in successful implementation of the PUPCB (or any intervention). Many nurses stated that for future implementation, education sessions or inservices would be needed to introduce the bundle to staff, familiarise them with what it involves and train them in its use. Nurses thought ideally, these sessions should be frequent, brief and concise; and involve all staff on the ward. The importance of providing evidence to nurses around the effectiveness of the PUPCB and the benefits of patient participation in care was also emphasised. They thought nurses need to view the intervention as necessary and important in order for it to be adopted in practice, and providing evidence was a way to ensure this.

*Providing some evidence about how much more the patients participate… doing an inservice on how pressure areas have gone down since the INTACT trial started, some hard evidence on the stuff. (Site 1, N3)*

#### Leadership and influence

Another important factor mentioned by nurses for implementing a PUPCB was leadership. A few nurses suggested there needs to be someone leading and driving implementation. This person was described as needing to be persistent, dedicated, having time to implement the intervention and probably being internal to the organisation so as to have some influence on staff. An external facilitator to provide staff education was also raised as being helpful during the INTACT trial. As lack of time and the risk of the intervention losing momentum over time were mentioned as potential barriers; having dedicated staff to facilitate implementation seemed important to nurses.

*You do need somebody to drive that responsibility or that role and it can’t just be something that you expect a ward to develop or to do. It has to be driven by somebody. (Site 2, N7)*

*….having an independent person to come in and that’s been their focus, to educate the staff. (Site 4, N16)*

Using hospitals’ key performance indicators (i.e. PUP and patient participation) to influence staff and show them the significance of the intervention was also mentioned as a strategy for implementation.

#### Keeping the PUPCB simple

In order for the intervention to work in practice, nurses raised a number of issues relating to the care bundle itself and processes of implementation. Many mentioned extra documentation was a major barrier and would hinder nurses from adopting the intervention; hence, they thought the intervention should not involve much paperwork.

*A tick-box on a care plan would be good… Not further reams of documentation. Nurses should be encouraged to feel that this conversation is easy to have, and necessary and not time consuming. (Site 1, N1)*

Nurses thought the intervention should be brief, simple and easy to deliver, in order to address another barrier frequently mentioned; lack of time. One suggested the DVD shown to patients as part of the PUPCB could be put on the patients’ television screens in hospital to save time. Several nurses thought the education should start pre-admission (for elective surgical patients) or on admission (for other patients) so that post-operatively, the message is just reinforced. Nurses said that as patients already received too much information post-operatively, receiving a message they had already heard would be easier to understand.

*When they come for their preoperative day about two weeks before surgery for knees and hips for example, it could perhaps be incorporated at that time. Before they start receiving their care when they’ve got time to think about it learn a bit more. (Site 3, N10)*

## Discussion

This study explored nurses’ perceptions of a PUPCB, as part of the process evaluation of a cluster randomised trial (INTACT) [13]. Overall, nurses had positive views and found the PUPCB feasible and acceptable. Their responses were represented in five themes: 1) Awareness of the PUPCB and its similarity to current practice; 2) Improving awareness, communication and participation with the PUPCB; 3) Appreciating the positive aspects of patient participation in care; 4) Perceived barriers to engaging patients in the PUPCB; and 5) Partnering with nursing staff to facilitate PUPCB implementation.

Whilst several systematic reviews of complex PUP interventions and PUP programs have been published [24, 25], we are unaware of any other process evaluations of PUP studies. The systematic reviews identify variable quality in previous trials; most are pre-post studies, and provide limited descriptions of intervention fidelity or implementation strategies [24, 25]. To our knowledge, none have assessed nurses’ perceptions of interventions or their views on implementation and sustainability. Previous studies suggest that acutely ill hospital patients want to be involved in their PUP care [5]; and this study shows that nurses support efforts to increase patient involvement and resources such as PUPCB can facilitate participation.

The positive response of nurses to a PUPCB promoting patient participation in PUP care may be partly due to the PUPCB being developed in collaboration with end users; that is, the views of patients and nurses were sought in a pilot study and their feedback was incorporated into the final design of the intervention [6, 20]. This is in line with an integrated knowledge translation approach, which is used to develop interventions that are likely to be relevant, acceptable and usable to end-users [26]. As the PUPCB reflected current clinical practice guidelines [27] and nurses found it acceptable in terms of understanding, using and seeing the benefits of it, the PUPCB may be a simple way to promote evidence-based practice and patient participation in care.

Translating research into routine clinical practice is recognised as a major challenge in health care [17]. The uptake of new interventions (innovations) may be affected by: (a) factors relating to the intervention itself and the evidence surrounding it; (b) the context in which it is implemented; and (c) the characteristics of adopters (clinicians) [17, 28, 29]. As most of these challenges revolve around how adopters perceive, understand, utilise, communicate, implement or sustain an intervention within a certain context, it is imperative to explore their perspectives of it before attempting implementation. The current study provides important insight for possible implementation of a PUPCB by exploring nurses’ experiences with and perceptions of the intervention.

Uptake of an innovation is likely to be affected by factors relating to the intervention itself and evidence surrounding it [17, 28]. A systematic review identified attributes of innovations that affect their uptake, which included the key elements of Rogers’ ‘Diffusion of Innovations’ theory [28, 30]. When adopters perceived an innovation to be advantageous; compatible with their values, norms and needs; simple to use; able to be experimented with; and the benefits were visible, innovations were more easily adopted and implemented [28]. In the current study, nurses acknowledged advantages of the PUPCB in terms of their perceived benefits to patients and nurses; improved awareness, communication and participation in care (i.e. relative advantage). Nurses seemed to respond more positively to the PUPCB when they thought it aligned with current ward or hospital initiatives such as patient participation in care and PUP; or when they personally agreed with the concept of patient-centred care used in the bundle (compatibility). The vast majority of nurses understood the PUPCB and thought it was easy to use; and they further highlighted the importance of low complexity in terms of implementation in the sub-theme ‘Keeping the PUPCB simple’. Trialability was discussed in terms of being able to adapt the PUPCB to patients with different needs, such as involving family members for patients who are cognitively impaired or allowing patients to participate to different extents. Finally, nurses described the importance of observability across several themes, discussing perceived benefits of the PUPCB in themes ‘Improving awareness, communication and participation with the PUPCB’ and ‘Appreciating patient participation in care’, and highlighting the importance of providing evidence to nurses around the effectiveness of the PUPCB in ‘Strategies for implementing a PUPCB’.

According to the Promoting Action on Research Implementation in Health Services (PARIHS) framework, successful implementation of research depends on the dynamic and simultaneous relationships between evidence, context and facilitation [29]. Credible evidence from high quality research studies, as well as from clinical and patient experiences, is likely to influence implementation and adoption [29]. In this study, nurses expressed their desire for evidence on the effectiveness of the PUPCB and of patient participation in care; they thought that providing nurses with this evidence would facilitate uptake of the PUPCB. Another study found that nurses desired learning opportunities for research utilisation, such as education and mentoring, to understand research findings and implement them in practice [31]. In terms of clinical experience, nurses in the current study reflected on the intervention by describing it and comparing it to current practices, as well as discussing the perceived effects and benefits of the intervention. Patient experience was also raised; nurses described how they thought the PUPCB changed patient behaviour and how and why they thought patients did or did not participate in PUP care.

The context in which a new innovation is introduced is also likely to have a large impact on implementation success [17, 28, 29]. Features of the health care organisation such as the absorptive capacity for new knowledge and a receptive context for change are positively associated with adoption of innovations [28]. Elements of these, which are consistent with the description of context in the PARIHS framework, include culture, evaluation and leadership [28, 29]. Nurses spoke about the strong culture of patient participation in their wards or hospitals; suggesting the philosophy of patient-centred care was influencing nurses at the front line. Participants explained how the PUPCB aligned with an existing culture of patient participation in care, consistent with Rogers’ theory around compatibility of interventions [30] and Greenhalgh’s ‘Innovation-system fit’, whereby an innovation that fits with the organisation’s existing values, norms, strategies and goals is more likely to be successfully implemented [28].

The organisation’s capacity to evaluate the innovation, by monitoring the change; evaluating its impact; and feeding results back to those involved, affects assimilation and sustainment [28]. In the current study, nurses requested feedback from the trial about the effectiveness of the care bundle for PUP in their wards, suggesting this would help facilitate uptake of the intervention. To some extent, nurses also evaluated the intervention themselves in terms of benefits for patients and staff they perceived. However, because this was a trial, feedback to nurses did not occur until after the study was completed. If the PUPCB was to be adopted in practice, it appears regular feedback would be important. Audit and feedback is one strategy that is moderately effective in changing practice [32].

Leadership [29], communication and influence [28] are imperative for implementing new innovations. Interpersonal communication and influence through social networks in a health care system is the most effective medium for promoting uptake of innovations [28]. This may be achieved by engaging individuals who have particular impact on their colleagues, such as opinion leaders, champions and boundary spanners [28]. This ties in with the concept of facilitation, that is, enablement of implementation of evidence into practice, which requires an individual with the appropriate roles, skills, knowledge and authority to apply evidence in practice [29, 33]. Nurses in the current study highlighted the need for a designated person to drive implementation of the PUPCB for successful adoption. This person, who would be labelled a facilitator in PARIHS [29], was perceived to ideally possess appropriate knowledge and skills and be in a suitable role to drive implementation.

Finally, the characteristics of those adopting or using the intervention are important determinants of its uptake. These may include individual clinicians’ knowledge, capability and motivation to use new evidence or innovations [28]. Individuals may also have different concerns at different stages of implementation [28]. Concerns in pre-adoption relate to awareness of an innovation, knowing what it does and how to use it, and understanding how it will affect them as users [28]. Nurses in the current study described their understanding of the PUPCB and that they became aware of it through attending inservices; their perceived benefits of the intervention; and barriers and enablers to its implementation and use in usual practice. Early involvement and commitment of staff at all levels during implementation increase the likelihood of success [28]. The findings of the current study related to implementation and maintenance of the PUPCB by adopters also resonate with Normalisation Process Theory. This theory considers coherence (meaning), cognitive participation (commitment), collective action (effort) and reflexive monitoring (comprehension) as important aspects of implementation [34, 35]. In the current study, nurses established coherence of the intervention by describing their understanding of its components and its similarities and differences to their usual practice. Cognitive participation in or commitment to the intervention seemed apparent through initiation, legitimation, enrolment and activation [34]. That is, most nurses agreed it should become part of routine practice, acknowledged the benefits of the intervention and described how they partnered with patients in PUP. Collective action or effort is the work required to organise and enact a practice [34]. Participants perceived that partnering with nurses, by communicating information and disseminating evidence on the PUPCB; having appropriate leadership and influence for facilitating implementation; and keeping the intervention simple and easy to deliver were key aspects of successful uptake of the PUPCB. Reflexive monitoring is considered after the intervention has been in place for some time, however nurses still suggested the results of the main trial be disseminated to staff in order for them to see it as valuable and advantageous; something that occurred several months after the study’s completion.

This was a relatively small study of 18 nurses from four Australian hospitals, so there is the possibility that the variation in all nurses’ views were not represented in our findings. However, we used maximum variation purposive sampling to improve generalisability; continued data collection until saturation was reached; and included both public and private hospitals across two states. These strategies may have increased the relevance of our findings for other similar settings. Secondly, the study included an evaluation of one particular PUPCB. While this may make the findings specific to this particular PUPCB, the findings may have applicability for the implementation and use of other multi-component PUP strategies [24, 25, 36].

## Conclusions

This study found that nurses responded positively to a PUPCB involving nurse-patient partnership to promote patient participation in care. Nurses saw the bundle as beneficial to both patients and nurses through improved awareness, communication and participation in care related to PUP. They found the PUPCB easy to understand, use and implement; however acknowledged barriers to engaging some patients in PUP care. Participants suggested that partnering with nursing staff through communication and dissemination, leadership and influence, and keeping the PUPCB simple were crucial for facilitating its implementation. As the PUPCB reflects current clinical practice guidelines and nurses in this study found the PUPCB was acceptable, its use may be an effective strategy for promoting evidence-based PUP care that is easily and cheaply implemented.

## Abbreviations

PU: Pressure ulcer
PUP: Pressure ulcer prevention
PUPCB: Pressure ulcer prevention care bundle
